# Interim results of an ongoing study on the use of non-invasive hemodynamic monitoring with Nexfin in critically ill patients

**DOI:** 10.1186/cc12128

**Published:** 2013-03-19

**Authors:** K Van de Vijver, V Brabers, C Pigozzi, L Vervliet, V Vanbiervliet, H Maes, I Vos, M Peetermans, N Van Regenmortel, I De laet, K Schoonheydt, H Dits, M Malbrain

**Affiliations:** 1 Ziekenhuis Netwerk Antwerpen, ZNA Stuivenberg, Antwerp, Belgium

## Introduction

The Nexfin monitor (BMEYE, the Netherlands) enables continuous non-invasive analysis of blood pressure (MAP) as well as cardiac output (CO) measurements. The aim of the present study was to validate the Nexfin in a mixed population of medical ICU patients.

## Methods

Interim results of a prospective ongoing study in 77 patients admitted to the medical ICU (46 patients mechanically ventilated, M/F ratio 1/1). Age 65.6 ± 15.9, BMI 25.6 ± 4.8, APACHE II score 22.9 ± 10.9, SAPS II 48 ± 20.1, SOFA score 7.5 ± 4.5. A modified outreach score (SOS) was calculated on admission. For all patients, simultaneous recording of arterial pressure by radial line (*n = *78), PiCCO (*n = *44) or by NIBP with arm cuff (*n = *47) was compared with the Nexfin monitor. Statistical analysis was performed with Student's *t *test, Pearson correlation and Bland-Altman analysis.

## Results

A total of 103 measurements in 77 patients were performed. In seven patients measurement with Nexfin was not possible. For CO (55 paired measurements), values were 6 ± 2.1 l/minute (range 2.6 to 12). Pearson's correlation coefficient comparing Nexfin-CO with reference CO showed a good correlation (*R*^2 ^= 0.52). Bland-Altman analysis comparing both CO techniques revealed a mean bias ± 2SD (LA) of 0.3 ± 3.6 l/minute (58% error). The MAP was 84.2 ± 15.6 mmHg (53 to 131.5) and values obtained with the Nexfin correlated well with the reference method with an *R*^2 ^of 0.72. Bland-Altman analysis comparing both MAP techniques revealed a mean bias ± 2SD (LA) of -0.3 ± 18 mmHg (20.9% error). However, Nexfin-MAP did not correlate well with NIBP (*R*^2 ^= 0.36). Hemoglobin values obtained with Nexfin Massimo technique did not correlate well with laboratory values (*R*^2 ^= 0.26, 33% error). The 26 patients that died in the ICU had higher APACHE II (*P *= 0.017), SAPS II (*P <*0.0001), SOFA (*P <*0.0001) and SOS (*P *= 0.004) scores and significantly lower MAP (*P <*0.0001), hemoglobin (*P *= 0.01) and lower dp/dtmax (*P *= 0.003), a marker for contractility. There were no outcome differences with regard to subgroup analysis in patients with either low or high CO or SVR.

## Conclusion

In unstable critically ill patients, MAP (and CO) can be monitored with the Nexfin. The exact patient population for this technology has yet to be defined and more patients are probably needed for pattern recognition, although the results indicate that low MAP and dp/dtmax are associated with poor outcome. In the future, Nexfin data could theoretically be incorporated in a new outreach score.

**Table 1:** Results of Bland and Altman analysis

Pulsioflex	AutoCal	PE (CCl) (%)	*n*	*R* ^2^	PE (TD-Pi) (%)	*n*	*R* ^2^	PE (TD-Pu) (%)	*n*	*R* ^2^
All	All	37.9	940	0.73	22.8	382	0.88	38.5	382	0.66
All	Yes	43.4	510	0.50	20.4	210	0.88	42.3	210	0.47
All	No	27.8	430	0.83	25.6	172	0.85	32.5	172	0.74
Fem	All	30.6	464	0.73	20.2	192	0.88	33.0	192	0.66
Rad	All	44.2	476	0.58	25.2	190	0.85	43.7	190	0.56

